# Supplementary material for: Estimating the course of the COVID-19 pandemic in Germany via spline-based hierarchical modelling of death counts
Source: Sci Rep. 2022 Jun 13;12:9784. doi: 10.1038/s41598-022-13723-y (PMC9191534; doi:10.1038/s41598-022-13723-y)
Supplement: Supplementary file 1 — Supplementary Information. [file 41598_2022_13723_MOESM1_ESM.pdf]

Supplement to "Estimating the course of the COVID-19 pandemic in Germany via spline-based hierarchical modelling of death counts"

Tobias Wistuba, Andreas Mayr and Christian Staerk

Department of Medical Biometry, Informatics and Epidemiology, University Hospital Bonn, Germany

S.1 Complete model formulations

Here, we provide complete formulations of the original Bayesian hierarchical model of Flaxman et al. [1] and of our adapted model which additionally incorporates splines as well as time-varying effective infection fatality rates (IFRs).
The original Bayesian hierarchical model of Flaxman et al. [1] is given as follows (using slightly adjusted notation to ensure direct comparability to our approach):

Reported deaths:	$D_{t,m} \sim \mathrm{NB}\Big(d_{t,m},\, d_{t,m} + \frac{d_{t,m}^2}{\psi}\Big),\ t = 1, 2, \ldots, \quad \psi \sim \mathcal{N}^+(0,\, 5)$
Expected deaths:	$d_{t,m} = \mathrm{IFR}_m^* \cdot \sum_{\tau=1}^{t-1} \pi_{t-\tau,m} I_{\tau,m}$
Discretized ITD time:	$\pi_{s,m} = \int_{s-0.5}^{s+0.5} \pi(\tau) d\tau,\ s = 2, 3, \ldots, \quad \pi_{1,m} = \int_0^{1.5} \pi(\tau) d\tau$
Infection-to-death (ITD):	$\pi \sim \mathrm{Gamma}(5.1,\, 0.86) + \mathrm{Gamma}(17.8,\, 0.45)$
Time-constant IFR:	$\mathrm{IFR}_m^* \sim \widehat{\mathrm{IFR}}_m \cdot \nu, \quad \nu \sim \mathcal{N}(1,\, 0.1)$
Infections:	$I_{t,m} = \Big(1 - \frac{\sum_{i=1}^{t-1} I_{i,m}}{N_m}\Big) \cdot R_{t,m} \cdot \sum_{\tau=1}^{t-1} I_{\tau,m} g_{t-\tau},\ t = 7, 8, \ldots$
Discretized gen. time::	$g_s = \int_{s-0.5}^{s+0.5} g(\tau) d\tau, s = 2, 3, \ldots, \quad g_1 = \int_0^{1.5} g(\tau) d\tau$
Generation time:	$g \sim \mathrm{Gamma}(6.5,\, 0.62)$
Initial infections:	$I_{t,m} \sim \mathrm{Exp}\Big(\frac{1}{\lambda}\Big),\ t = 1, \ldots, 6, \quad \lambda \sim \mathrm{Exp}(0.03)$
Effective repr. number:	$R_{t,m} = R_{0,m} \cdot \exp\Big(-\sum_{k=1}^{6} \alpha_k \mathbb{1}_{k,t,m} - \beta_m \mathbb{1}_{t,m}^*\Big)$
Basic repr. number:	$R_{0,m} \sim \mathcal{N}^+(3.28, \lvert\kappa\rvert), \quad \kappa \sim \mathcal{N}(0,\, 0.5)$
Intervention priors:	$\alpha_k \sim \mathrm{Gamma}\left(\frac{1}{6},\, 1\right) - \frac{\log(1.05)}{6}$
Last intervention prior:	$\beta_j \sim \mathcal{N}(0,\, \gamma),\ j = 1, \ldots, M, \quad \gamma \sim \mathcal{N}^+(0,\, 0.2)$

In this model, the index parameter t refers to the t-th day of modelling and the index parameter m refers to the m-th country out of the M countries included in the model of Flaxman et al. [1]. The parameter k refers to the k-th non-pharmaceutical intervention (out of six considered interventions, see [1] for details). Furthermore, $R_{0,m}$ refers to the basic reproduction number for country m, N_m to the total population of country m, α_k to the effect of intervention k and β_m to the effect of the last considered non-pharmaceutical

intervention in country m. The estimated time-constant infection fatality rate for country m is denoted by IFR^*_m. The variables $\mathbb{1}_{k,t,m}$ and $\mathbb{1}_{t,m}$ indicate the implemented non-pharmaceutical interventions: if intervention k is active on day t in country m, then $\mathbb{1}_{k,t,m} = 1$, else $\mathbb{1}_{k,t,m} = 0$; similarly, if the last intervention of country m is active on day t, then $\mathbb{1}^*_{t,m} = 1$, else $\mathbb{1}^*_{t,m} = 0$. The given parameters of the gamma distributions are the mean and coefficient of variation. The interpretation of all other parameters is equivalent to the ones in our model, as described in the Methods section of the main paper. We refer to the supplementary material of the paper by Flaxman et al. [1] for a detailed description and discussion of their model.

Our spline-based Bayesian hierarchical model is given as follows:

Reported deaths:	$D_t \sim \text{NB}\Big(d_t,\, d_t + \frac{d_t^2}{\psi}\Big),\; t = 1, 2, \ldots, \quad \psi \sim \mathcal{N}^+(0,\, 5)$
Expected deaths:	$d_t = \sum_{\tau=1}^{t-1} \pi_{t-\tau} \cdot \widehat{\text{IFR}}_\tau \cdot \nu \cdot I_\tau, \quad \nu \sim \mathcal{N}(1,\, 0.1)$
Discretized ITD time:	$\pi_s = \int_{s-0.5}^{s+0.5} \pi(\tau) d\tau, s = 2, 3, \ldots, \quad \pi_1 = \int_0^{1.5} \pi(\tau) d\tau$
Infection-to-death (ITD):	$\pi \sim \text{Lognormal}(1.62,\, 0.42) + \text{Gamma}(17.8,\, 0.45)$
Time-varying eff. IFR:	$\widehat{\text{IFR}}_\tau = \frac{1}{C_w} \sum_{a \in A} C_{a,w} \cdot \widehat{\text{IFR}}_a,$ for day $\tau + 10$ in week w
Infections:	$I_t = R_t \cdot \sum_{\tau=1}^{t-1} I_\tau \cdot g_{t-\tau},\; t = 7, 8, \ldots$
Discretized gen. time:	$g_s = \int_{s-0.5}^{s+0.5} g(\tau) d\tau,\; s = 2, 3, \ldots, \quad g_1 = \int_0^{1.5} g(\tau) d\tau$
Generation time:	$g \sim \text{Lognormal}(1.39,\, 0.57)$
Initial infections:	$I_t \sim \text{Exp}\Big(\frac{1}{\lambda}\Big),\; t = 1, \ldots, 6, \quad \lambda \sim \text{Exp}(10)$
Effective repr. number:	$R_t = \max\Big(\sum_p a_p B_{p,3}(t),\, 0\Big)$
Spline coefficients prior:	$a_p \sim \mathcal{N}(a_{p-1},\, \theta),\; p > 1, \quad \theta \sim \mathcal{N}^+(0,\, 1)$
First spline coefficient prior:	$a_1 \sim \mathcal{N}^+(0,\, 1)$

The given parameters of the gamma distribution are the mean and coefficient of variation and the given parameters of the log-normal distribution are the mean and standard deviation of the underlying normal distribution.

Compared to the original model by Flaxman et al. [1], there are two crucial adaptations in our model: First, our model is able to account for time-varying effective infection fatality rates (IFRs) based on the changing age distribution of (confirmed) infections. Second, our model incorporates splines to estimate effective reproduction numbers without the need of prespecifying discrete change points based on adaptations in non-pharmaceutical interventions (NPIs). The two approaches also differ regarding their main target of analysis: While Flaxman et al. focus on estimating the effects of specific NPIs based on data from multiple countries, our spline-based model focuses on the smooth and data-driven estimation of the effective reproduction number for one particular country (or federal state). Apart from these important differences as well as additional slight adjustments regarding prior parameters based on recent literature [2, 3], the two modelling approaches are similar and follow the same hierarchical structure. We refer to the Methods section of the main paper for a detailed description of our model.

S.2 Effective infection fatality rate (IFR)

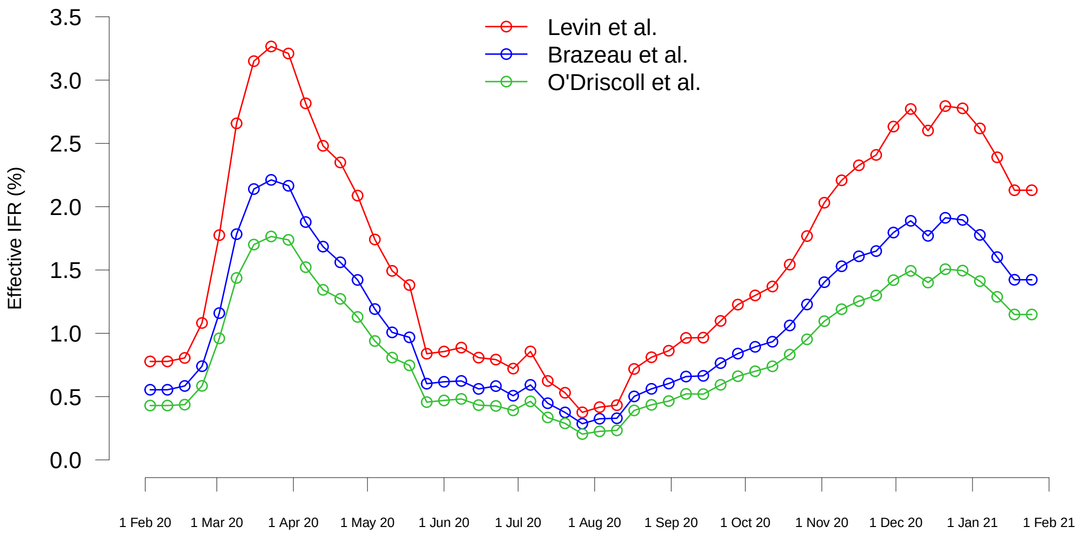

Figure S1: Effective infection fatality rate (IFR) estimates per calendar week for Germany, based on the three considered international studies providing age-specific IFR estimates (O'Driscoll et al. [4], Levin et al. [5], Brazeau et al. [6]).

S.3 Sensitivity analyses with alternative age-specific IFR estimates

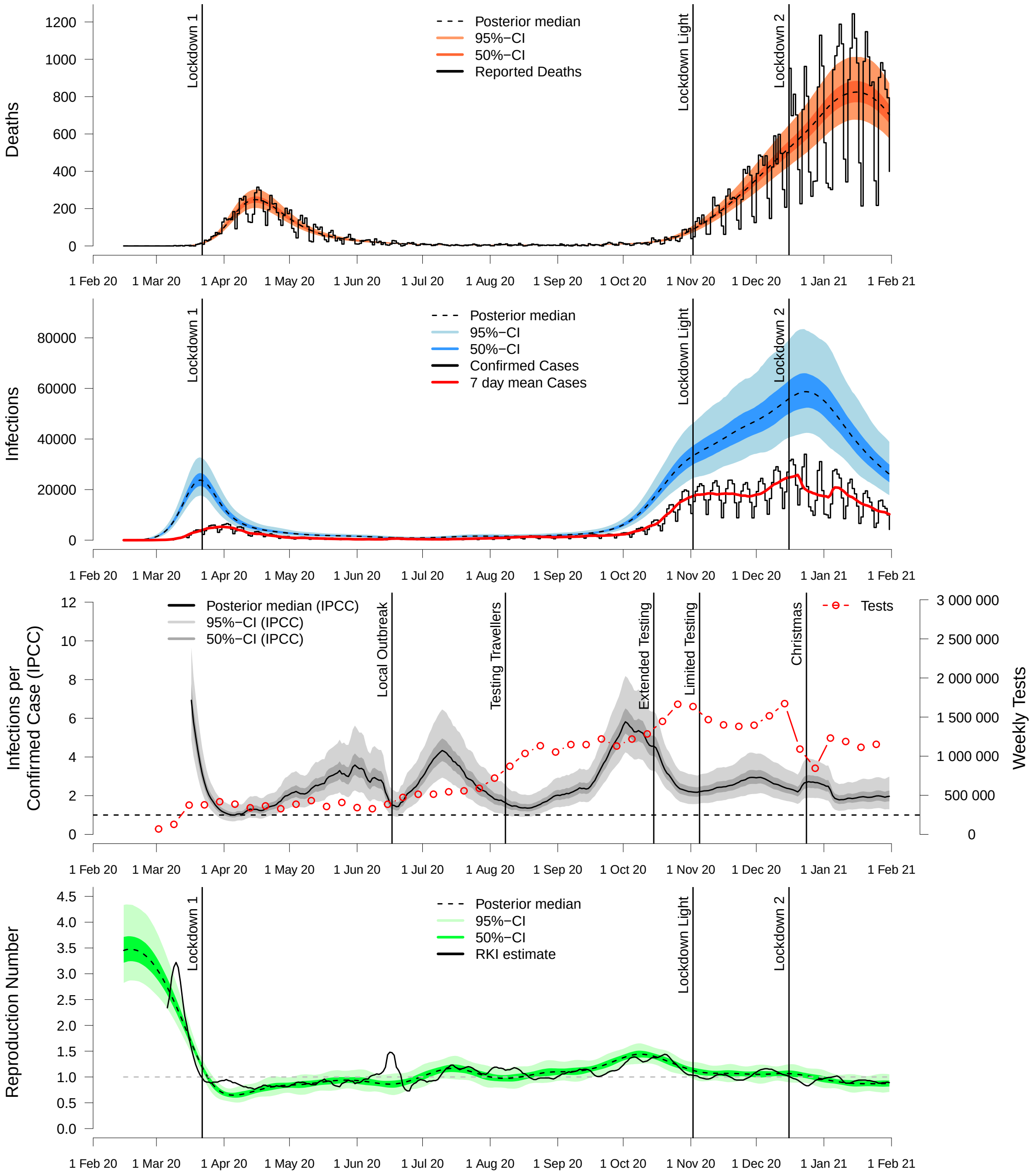

Figure S2: Results for Germany based on age-specific IFR estimates from O'Driscoll et al. [4].

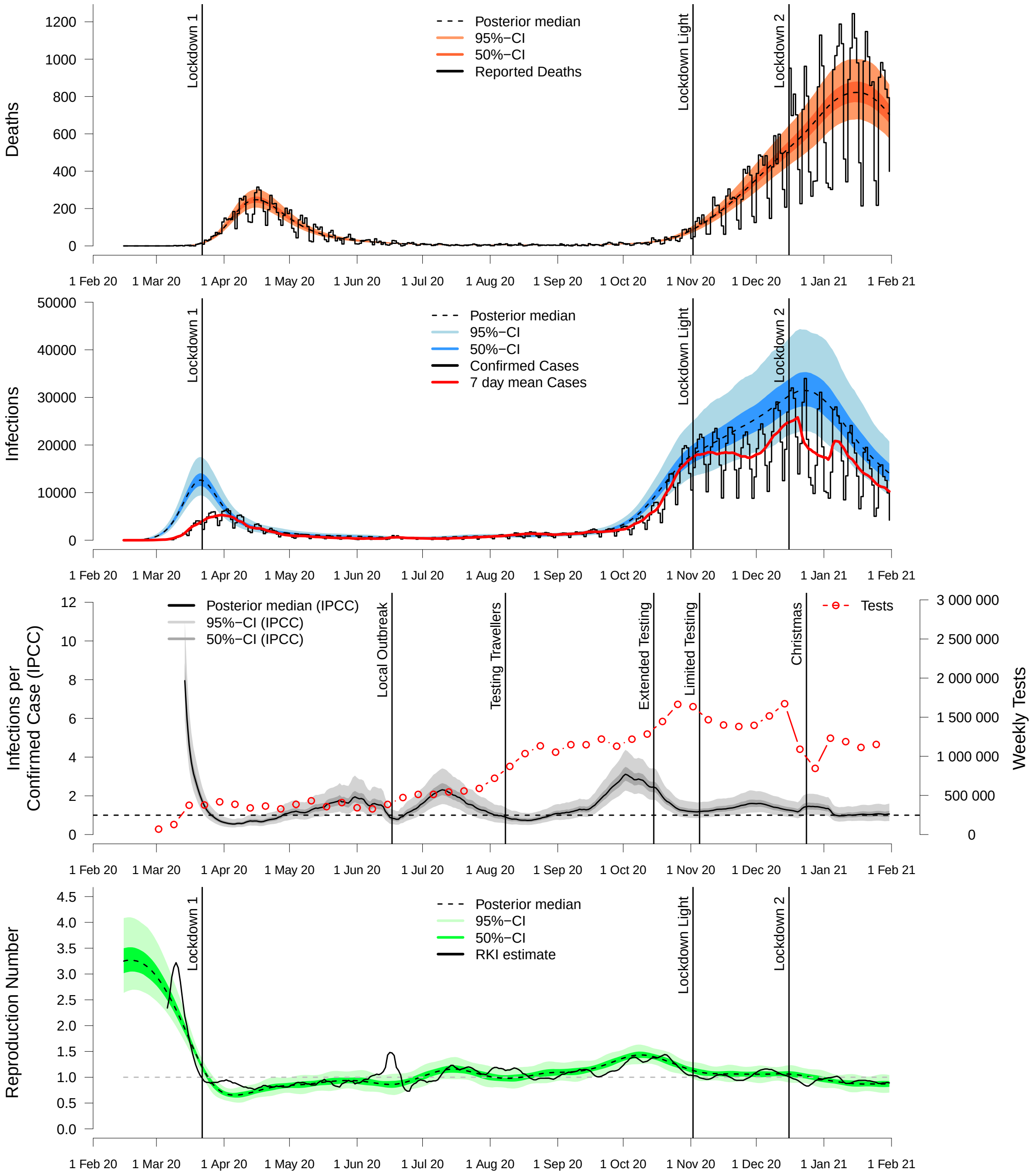

Figure S3: Results for Germany based on age-specific IFR estimates from Levin et al. [5].

S.4 Results for the 16 German federal states

S.4.1 Baden-Württemberg

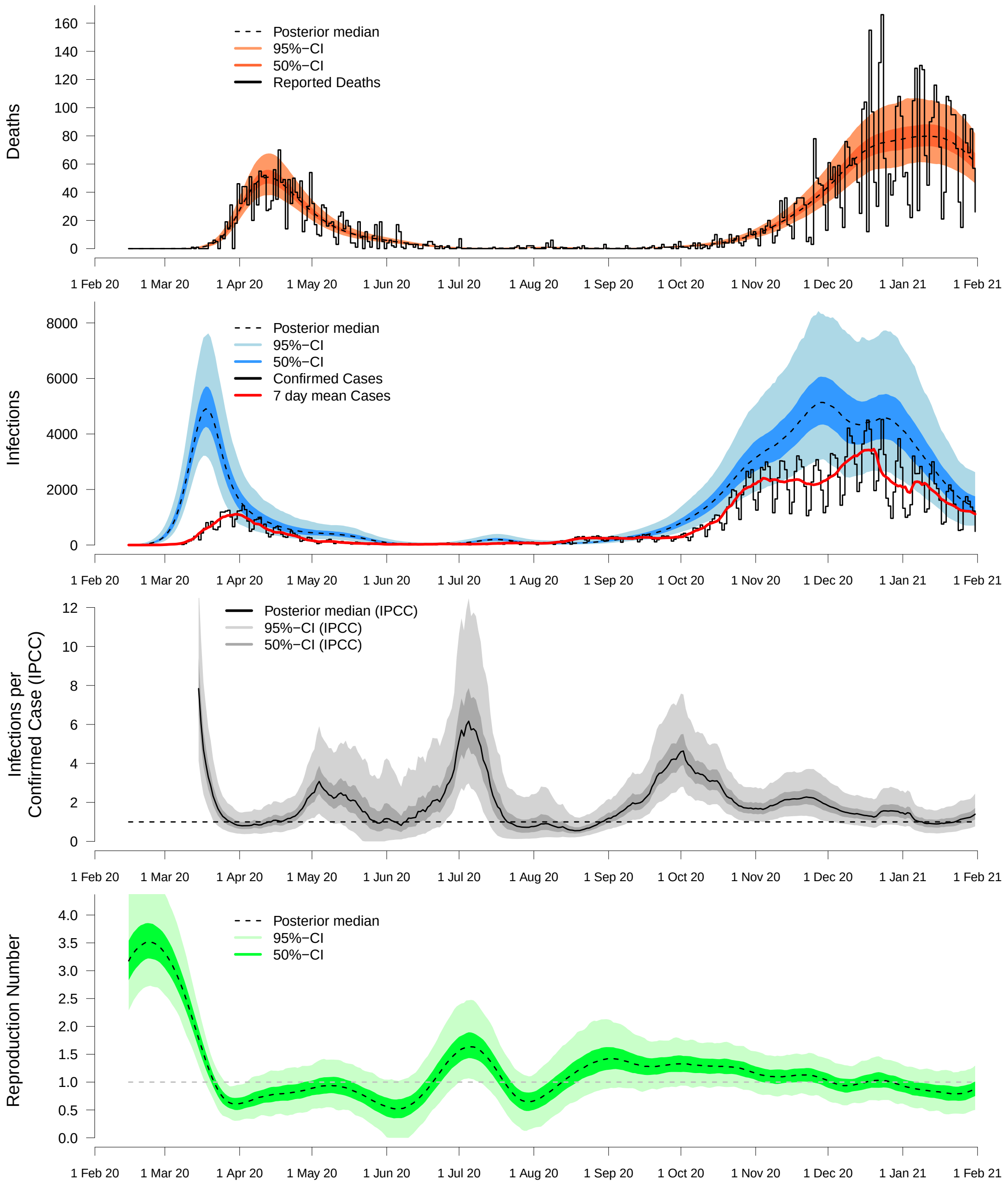

Figure S4: Results for Baden-Württemberg based on age-specific IFR estimates from Brazeau et al. [6].

S.4.2 Bavaria

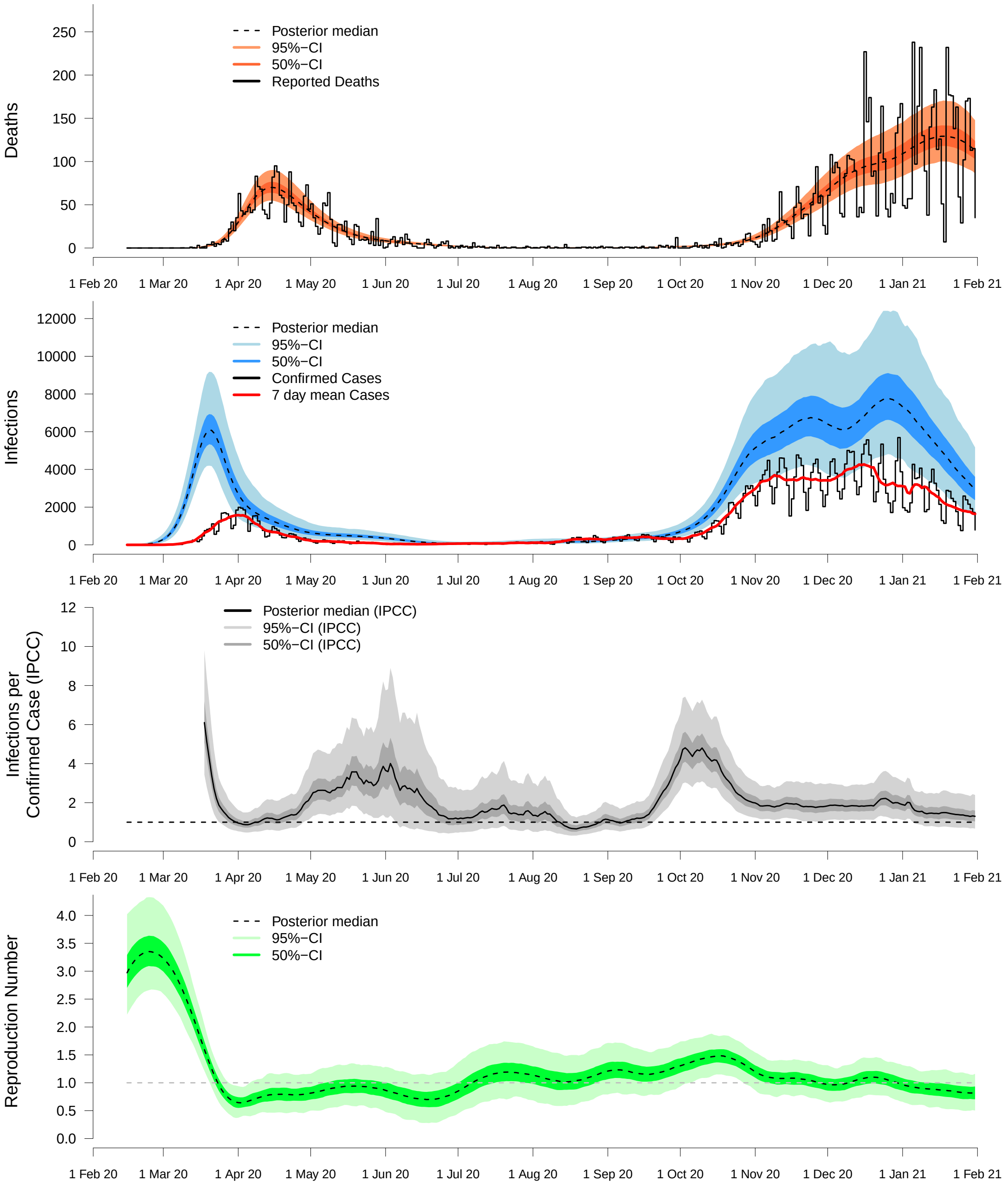

Figure S5: Results for Bavaria based on age-specific IFR estimates from Brazeau et al. [6].

S.4.3 Berlin

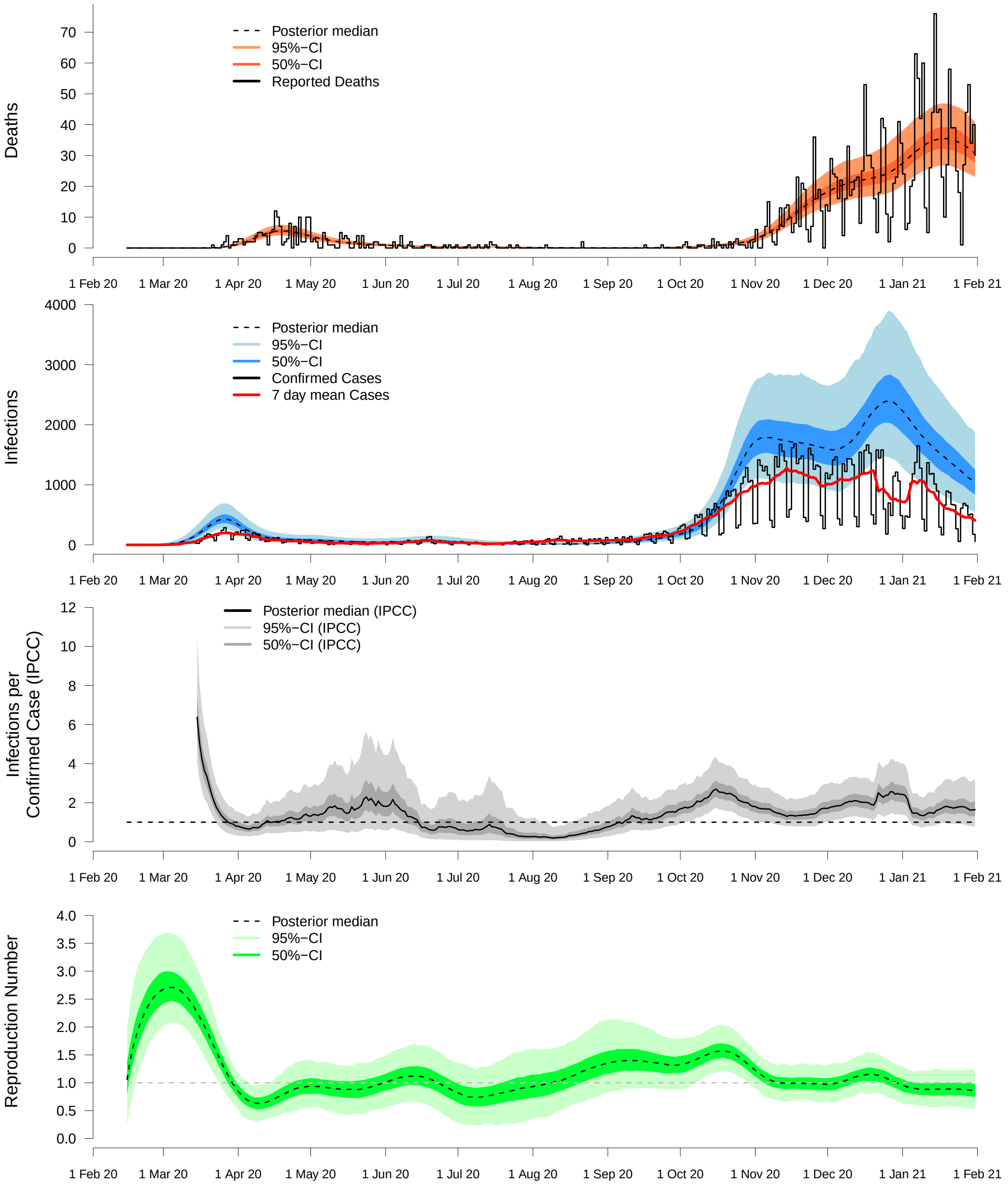

Figure S6: Results for Berlin based on age-specific IFR estimates from Brazeau et al. [6].

S.4.4 Brandenburg

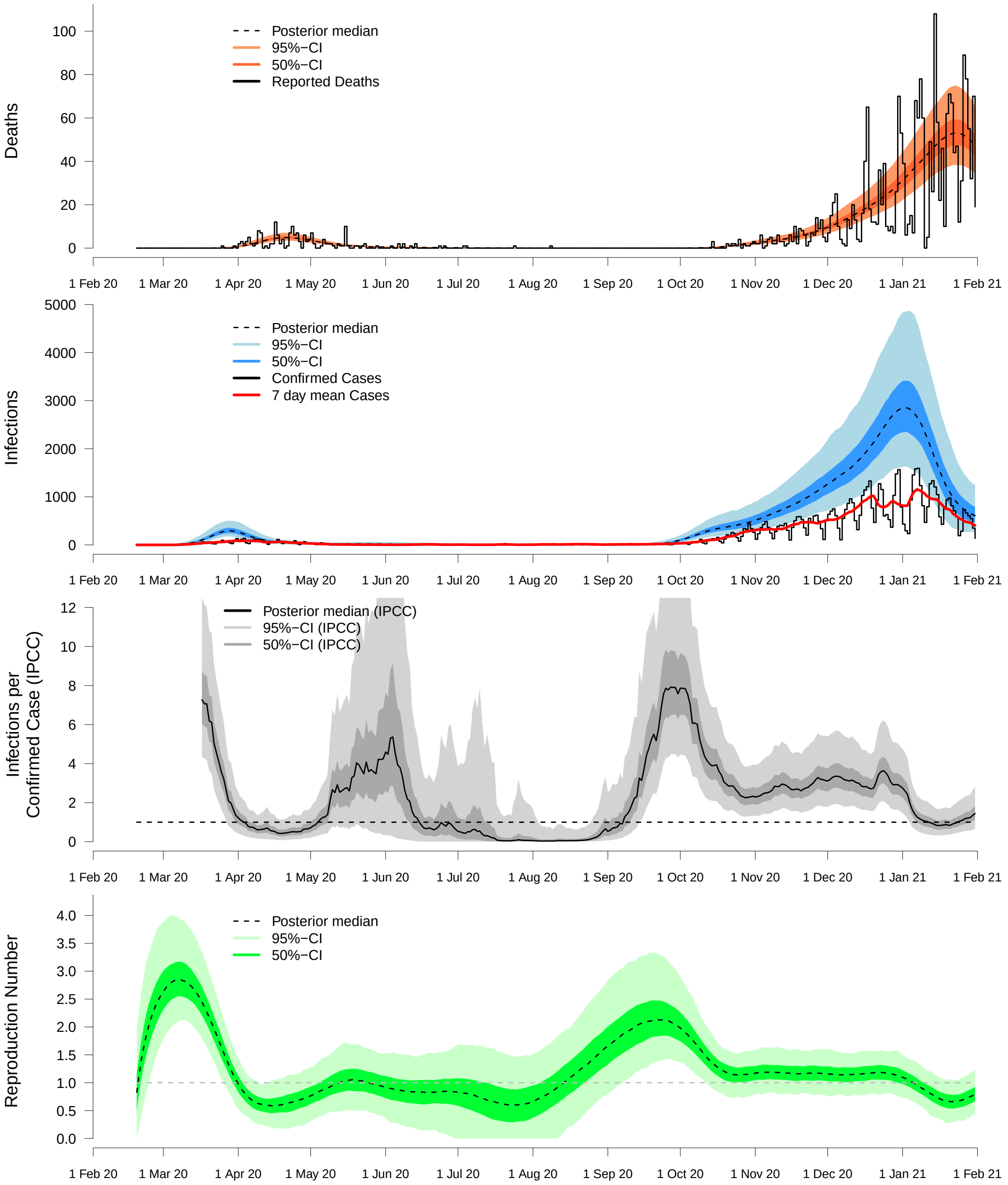

Figure S7: Results for Brandenburg based on age-specific IFR estimates from Brazeau et al. [6].

S.4.5 Bremen

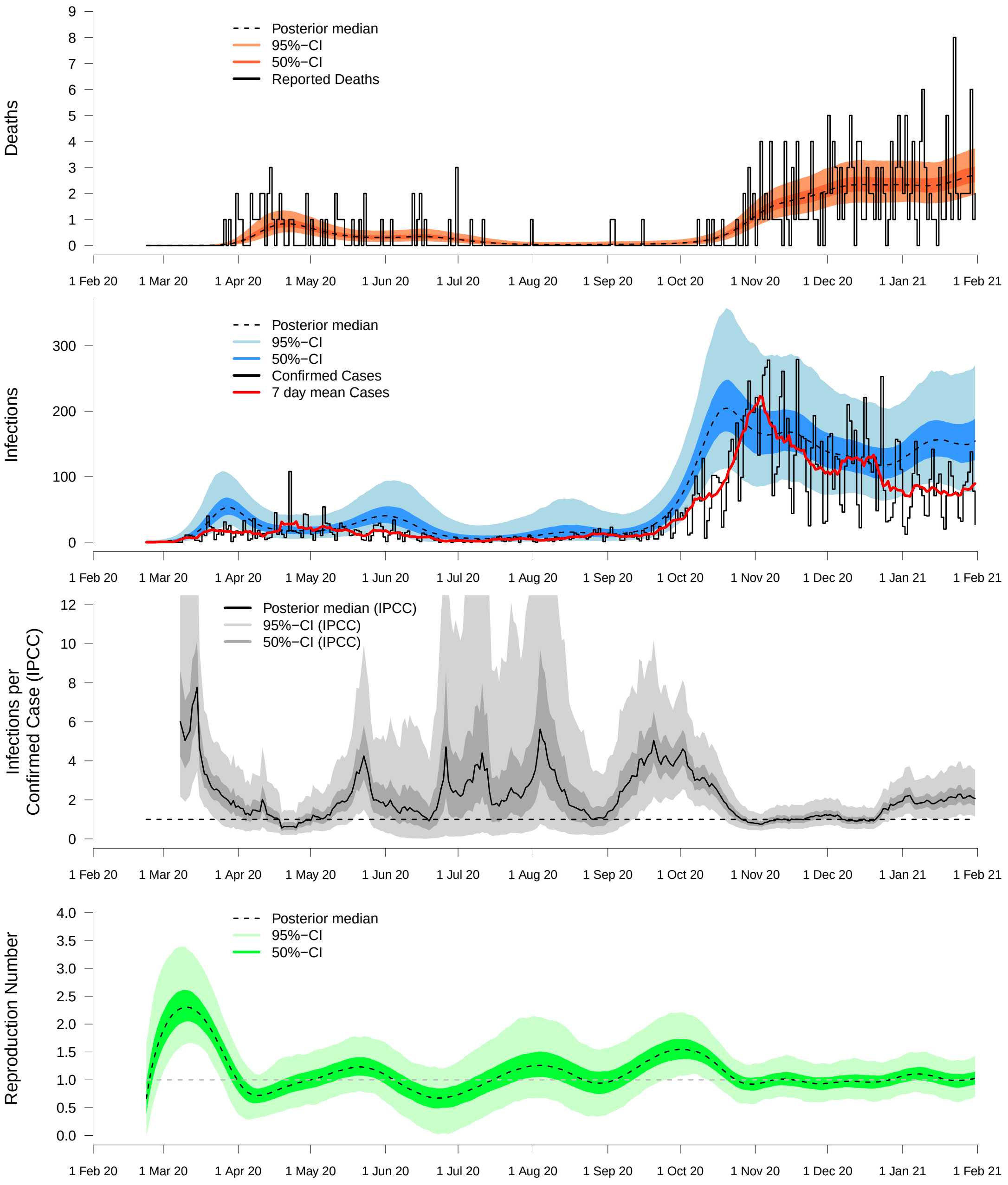

Figure S8: Results for Bremen based on age-specific IFR estimates from Brazeau et al. [6].

S.4.6 Hamburg

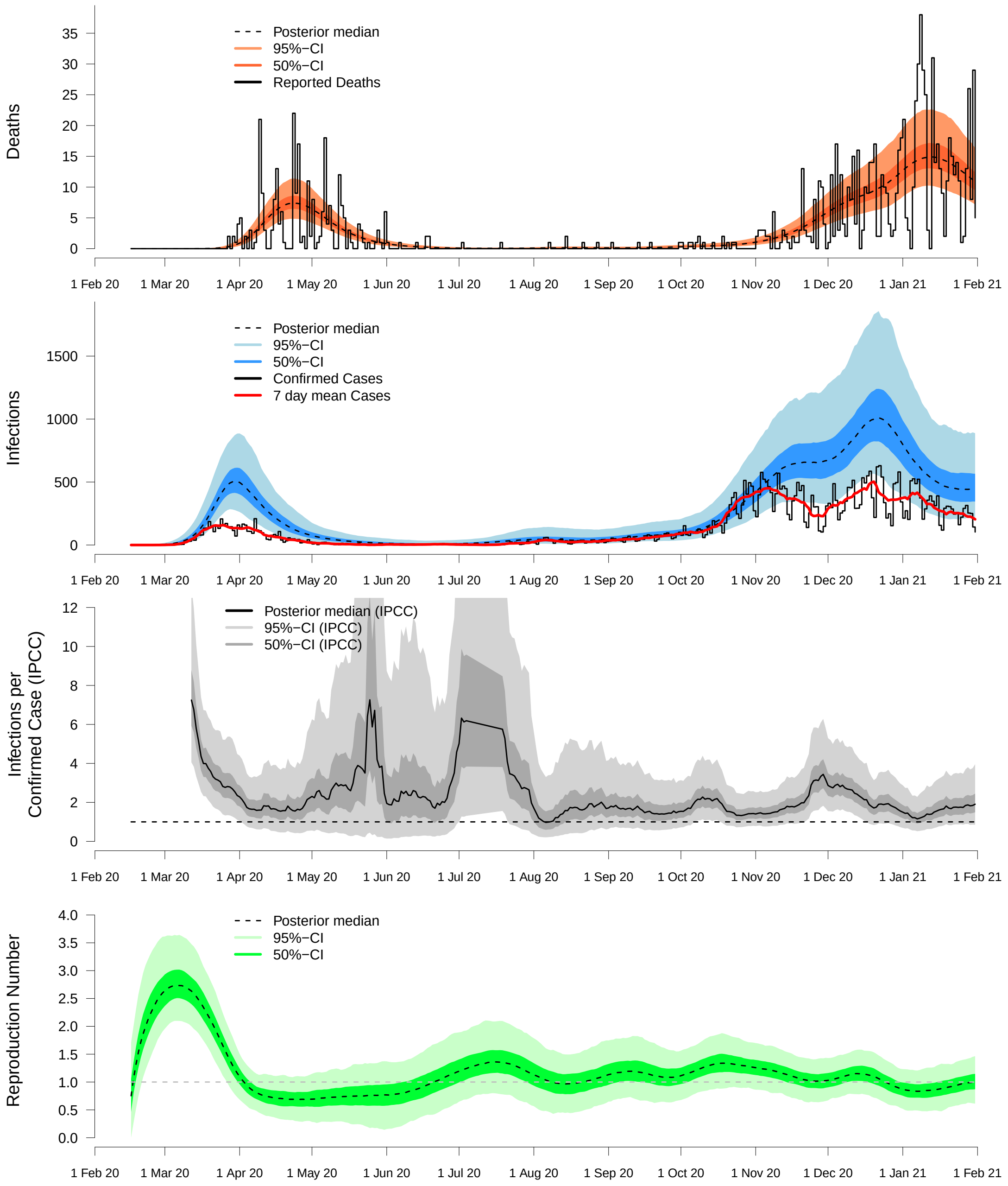

Figure S9: Results for Hamburg based on age-specific IFR estimates from Brazeau et al. [6].

S.4.7 Hesse

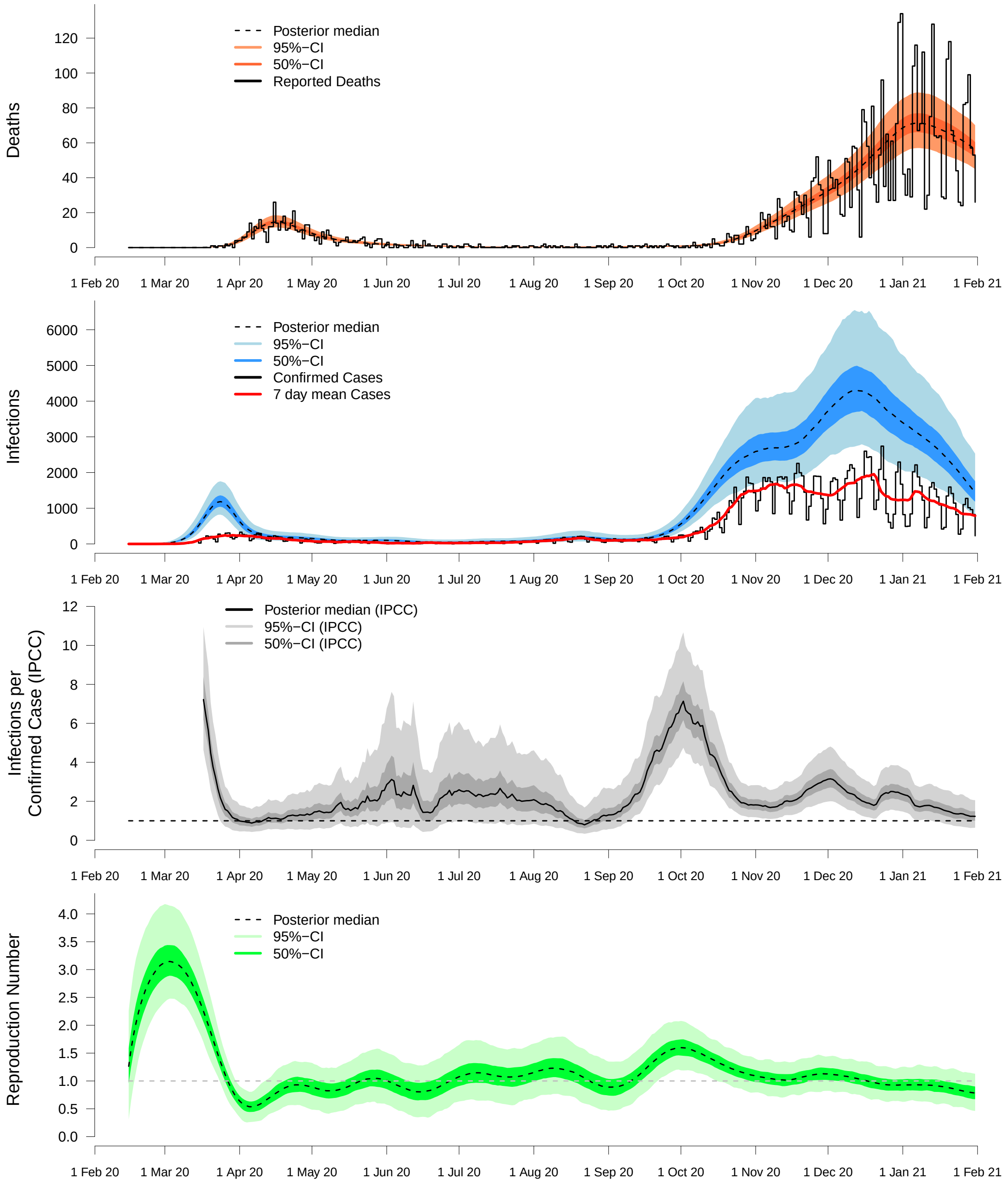

Figure S10: Results for Hesse based on age-specific IFR estimates from Brazeau et al. [6].

S.4.8 Mecklenburg-Western Pomerania

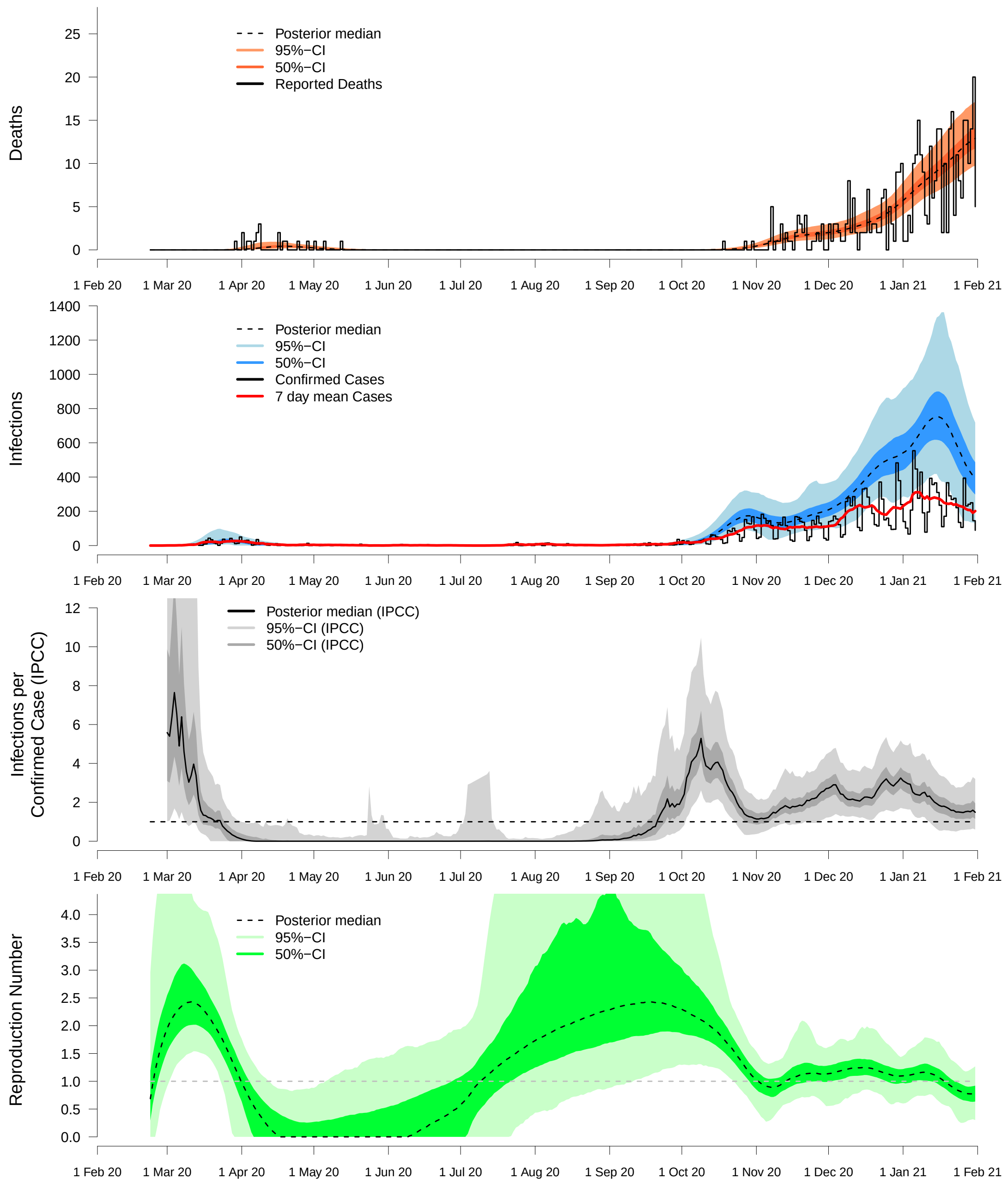

Figure S11: Results for Mecklenburg-Western Pomerania based on age-specific IFR estimates from Brazeau et al. [6].

S.4.9 Lower Saxony

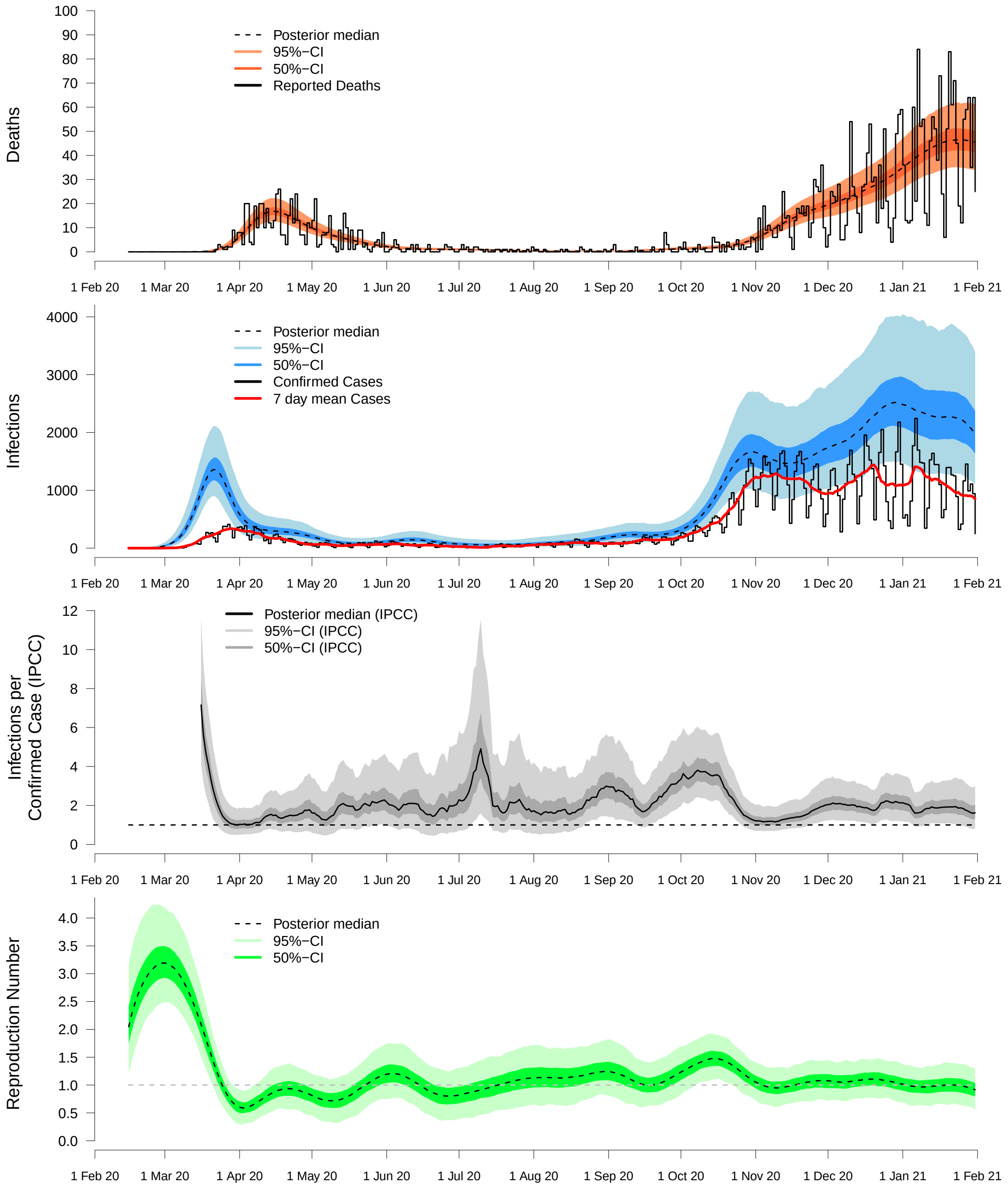

Figure S12: Results for Lower Saxony based on age-specific IFR estimates from Brazeau et al. [6].

S.4.10 North Rhine-Westphalia

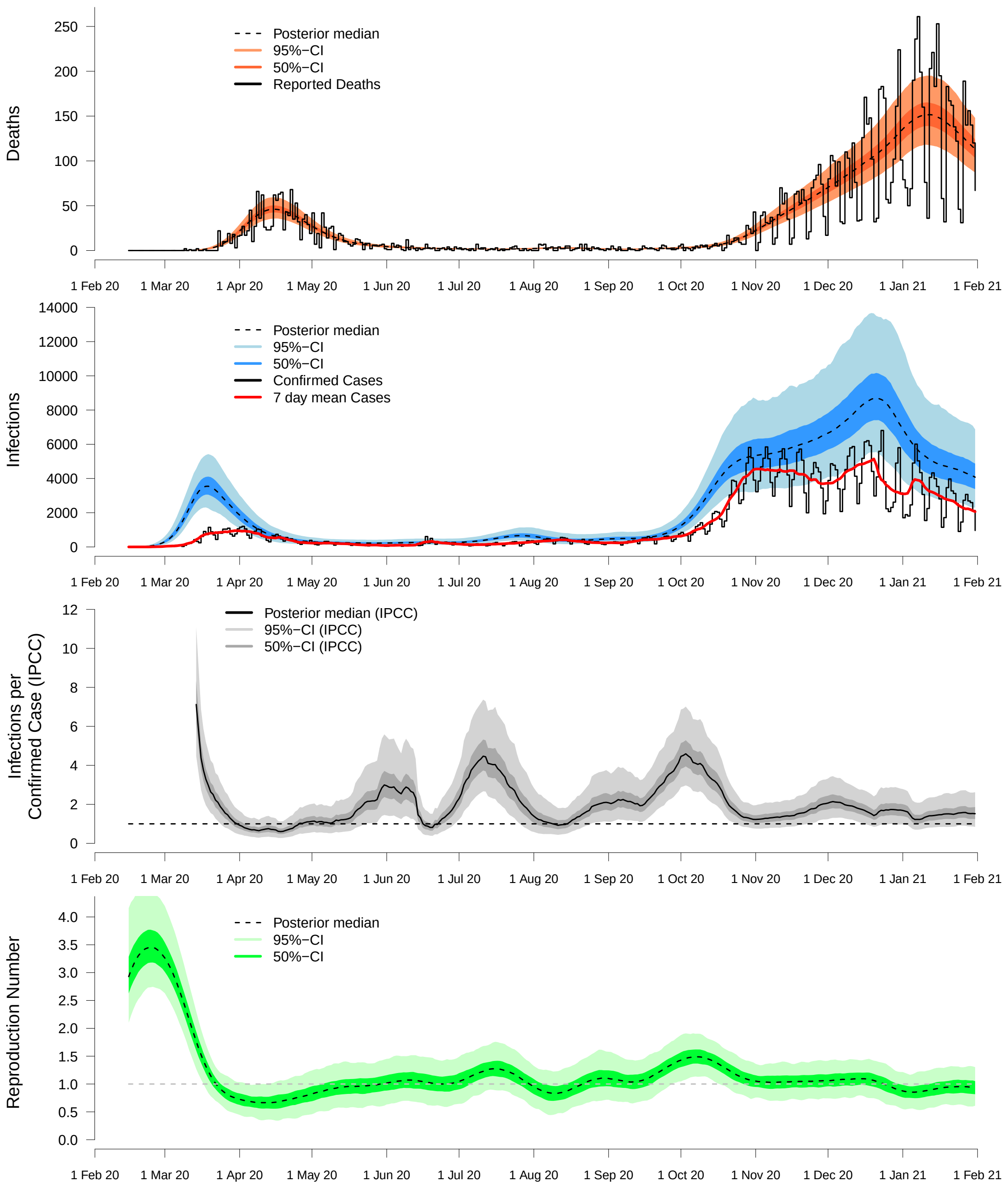

Figure S13: Results for North Rhine-Westphalia based on age-specific IFR estimates from Brazeau et al. [6].

S.4.11 Rhineland-Palatinate

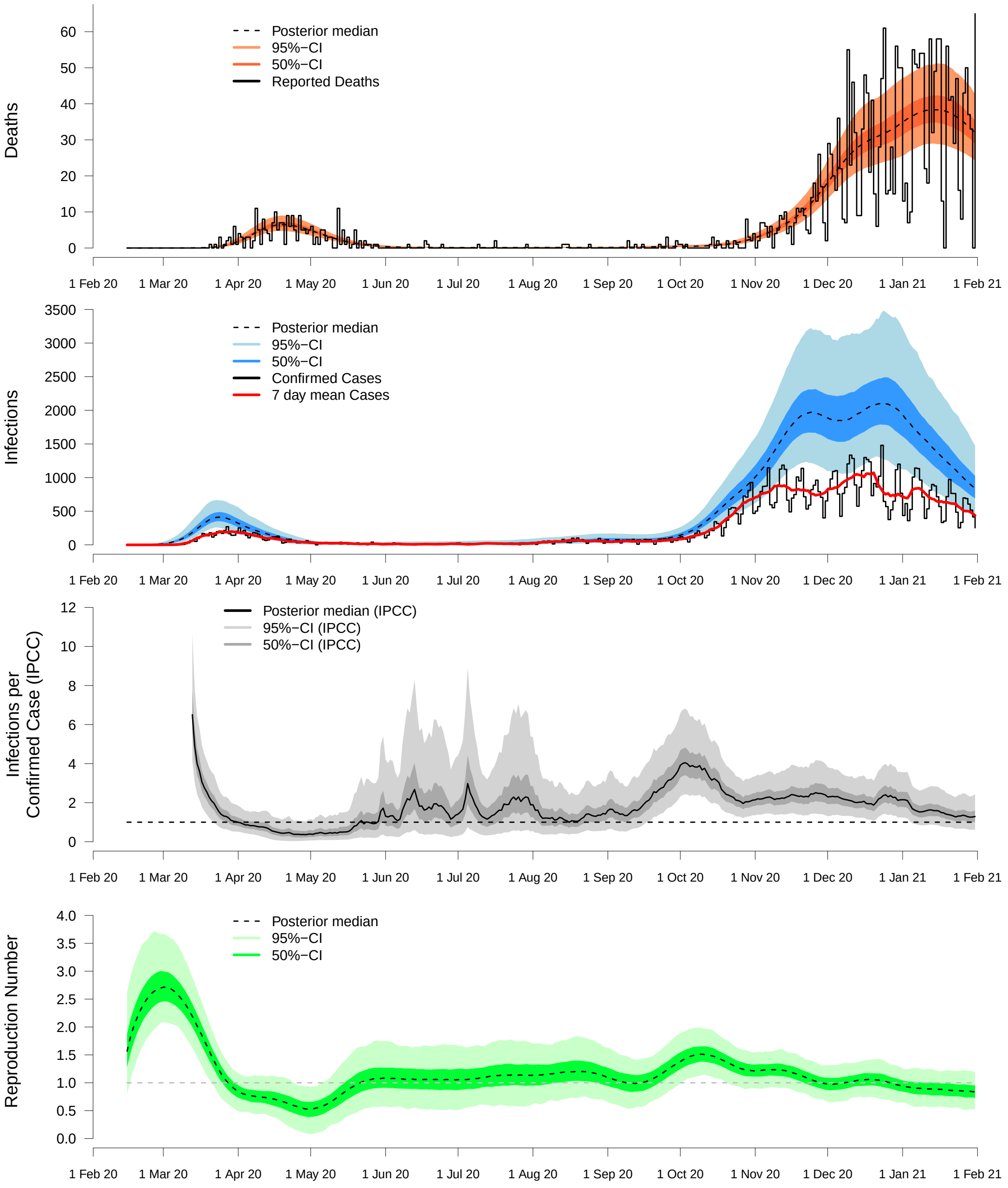

Figure S14: Results for Rhineland-Palatinate based on age-specific IFR estimates from Brazeau et al. [6].

S.4.12 Saarland

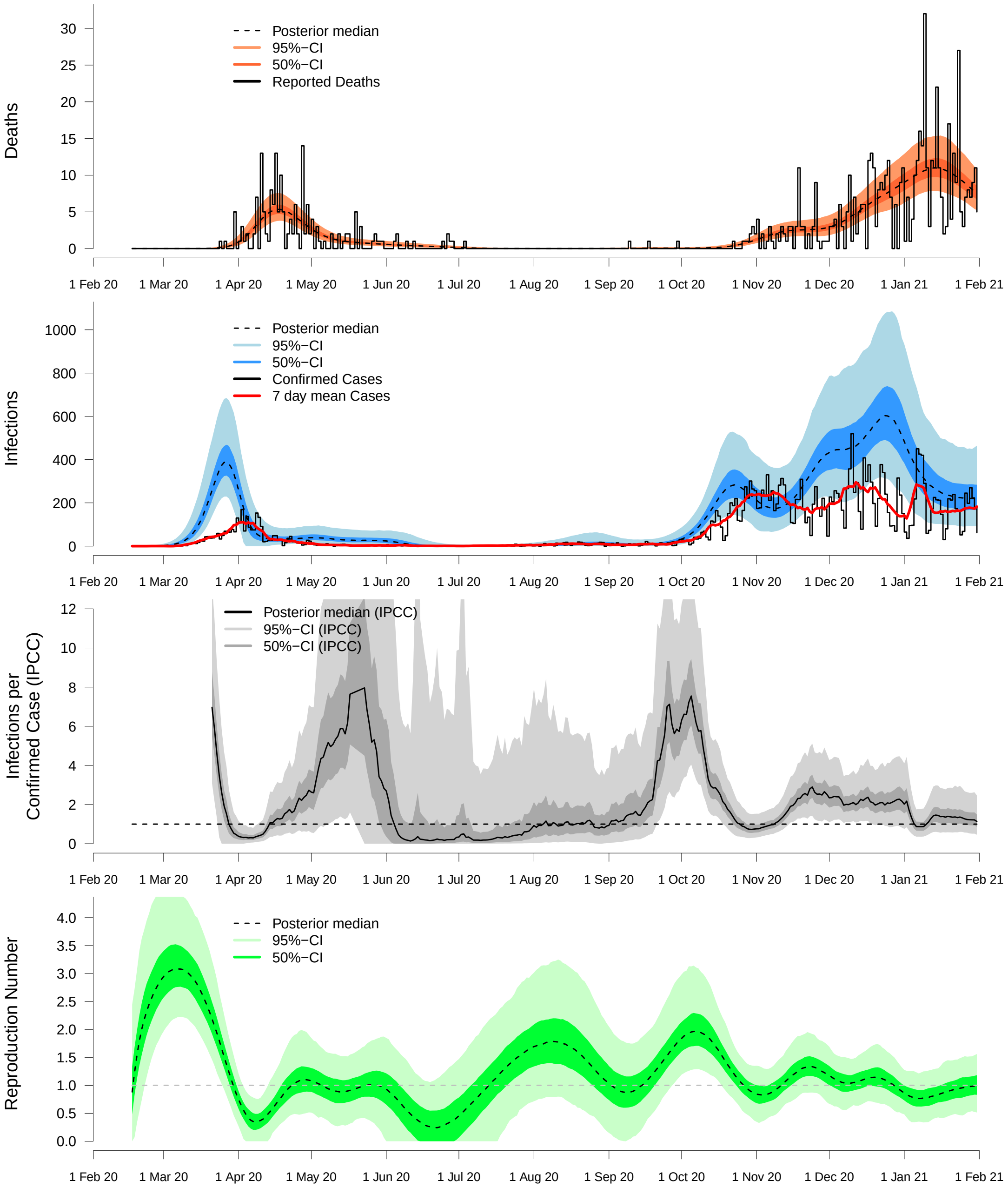

Figure S15: Results for Saarland based on age-specific IFR estimates from Brazeau et al. [6].

S.4.13 Saxony

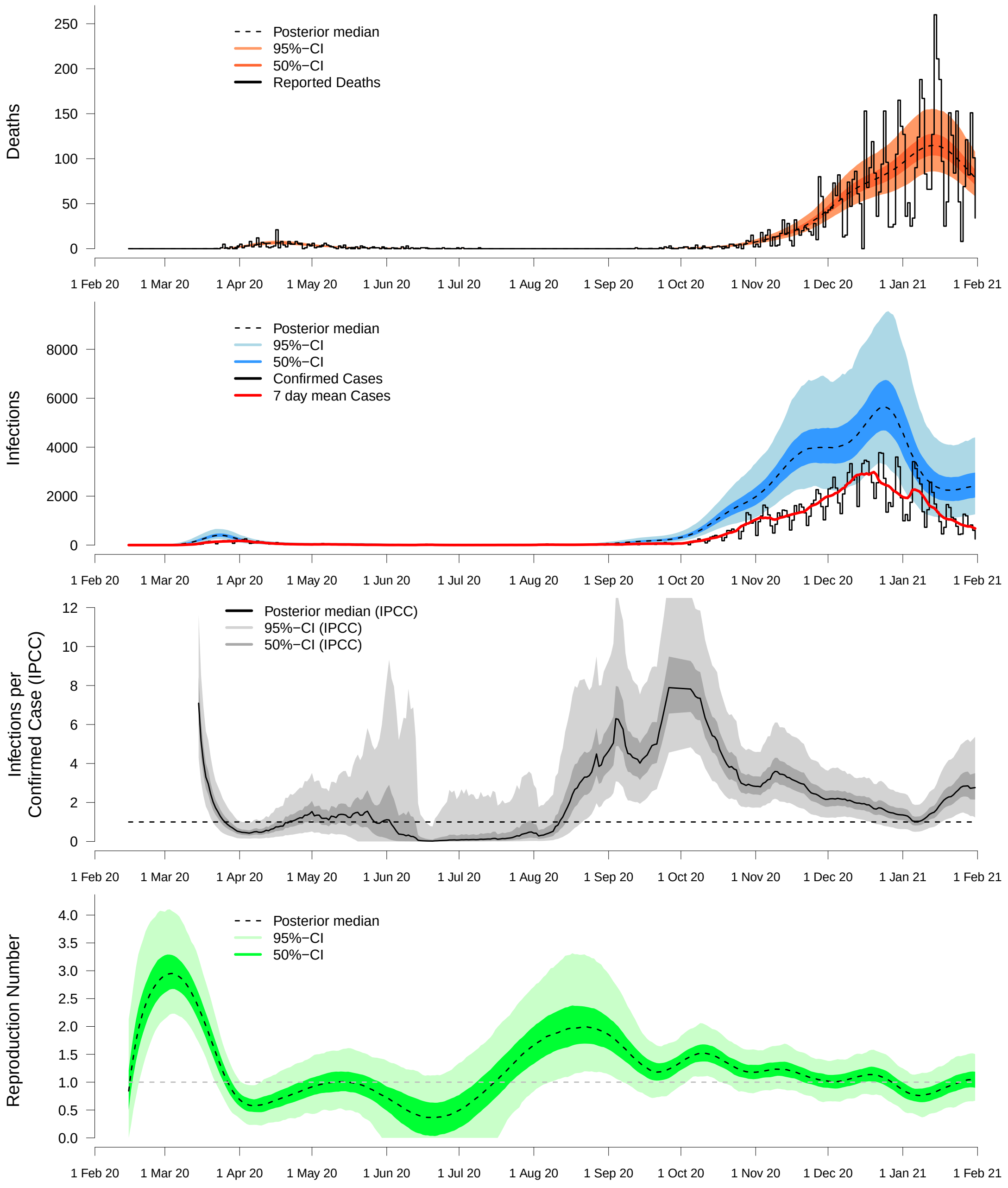

Figure S16: Results for Saxony based on age-specific IFR estimates from Brazeau et al. [6].

S.4.14 Saxony-Anhalt

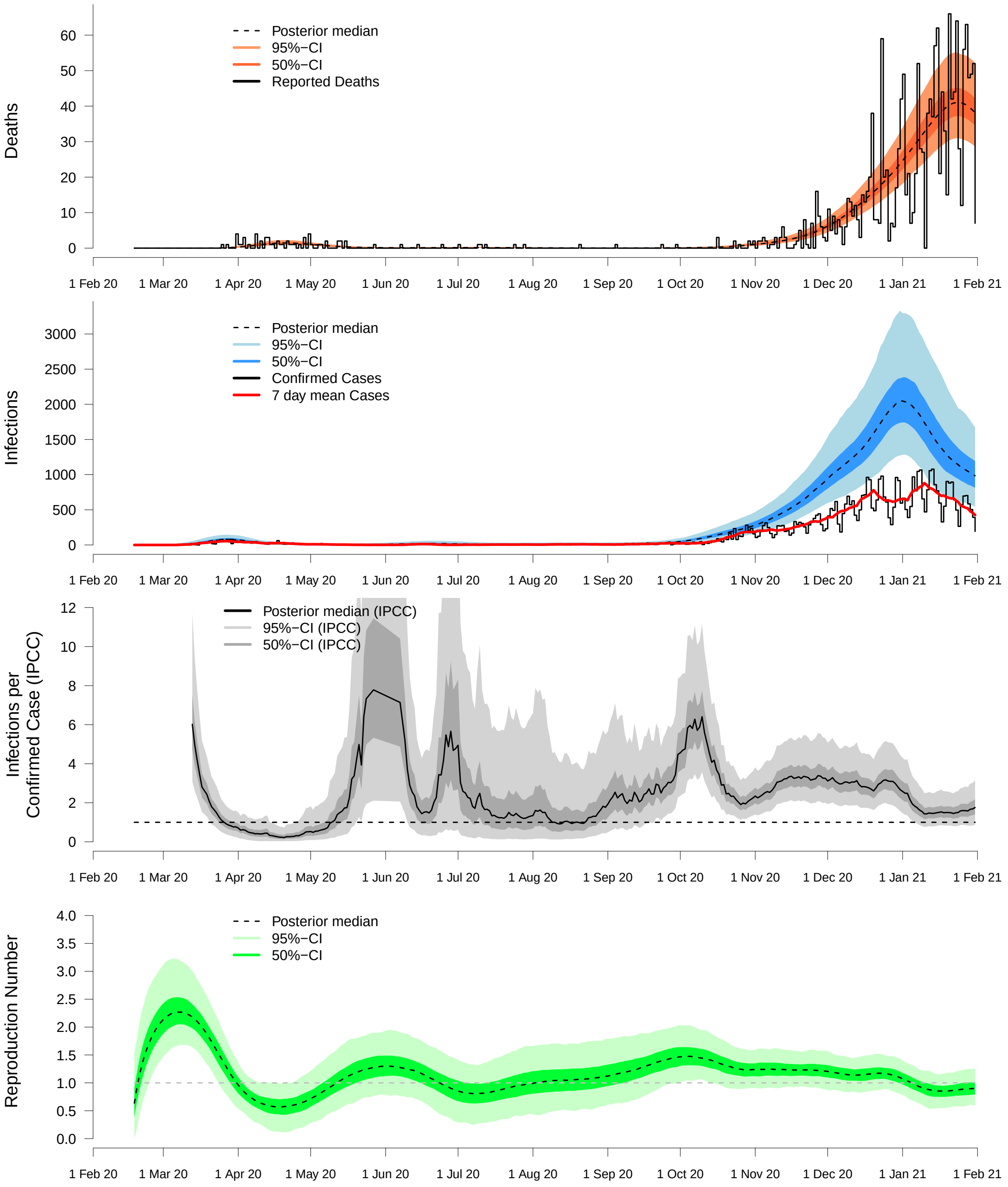

Figure S17: Results for Saxony-Anhalt based on age-specific IFR estimates from Brazeau et al. [6].

S.4.15 Schleswig-Holstein

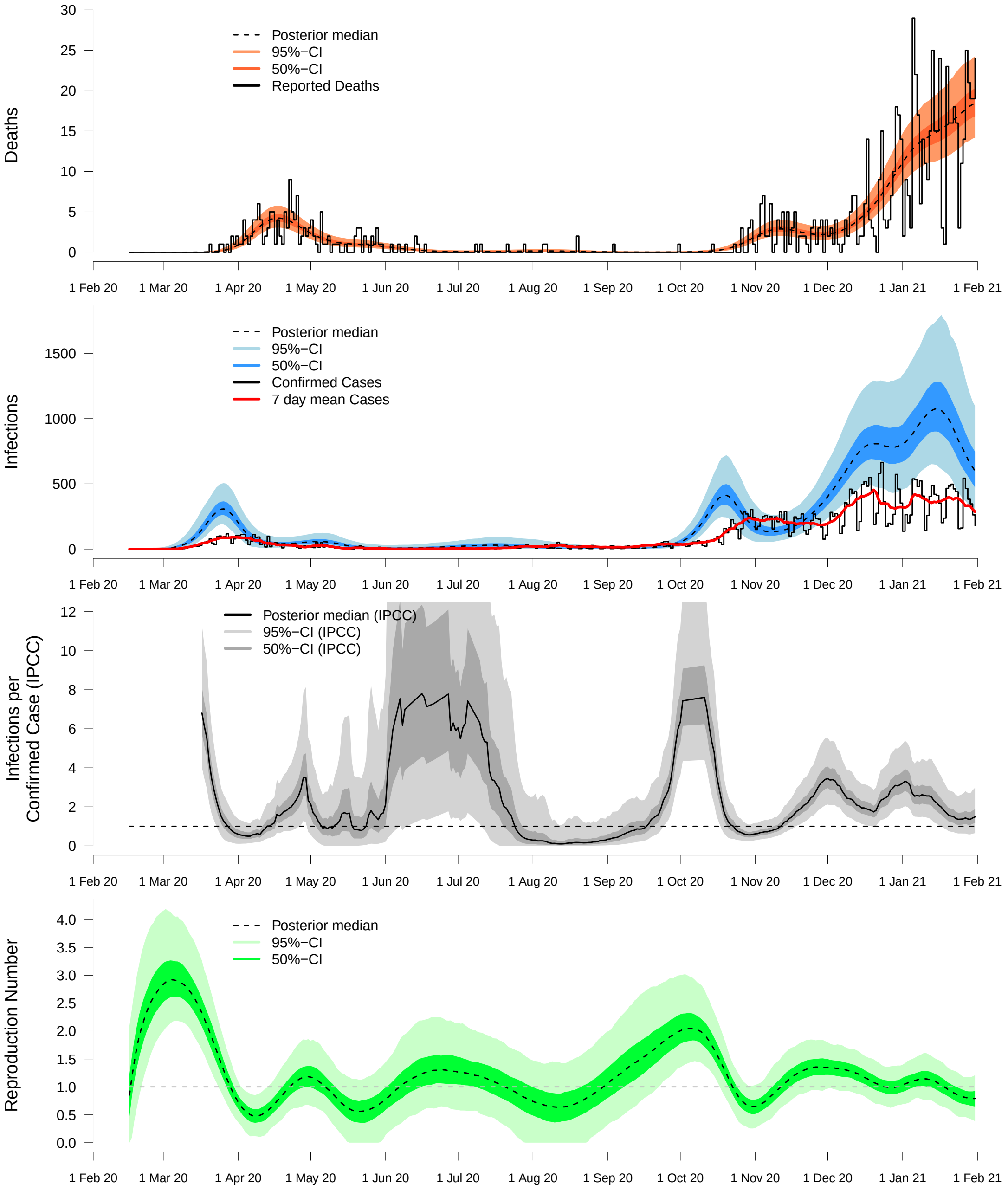

Figure S18: Results for Schleswig-Holstein based on age-specific IFR estimates from Brazeau et al. [6].

S.4.16 Thuringia

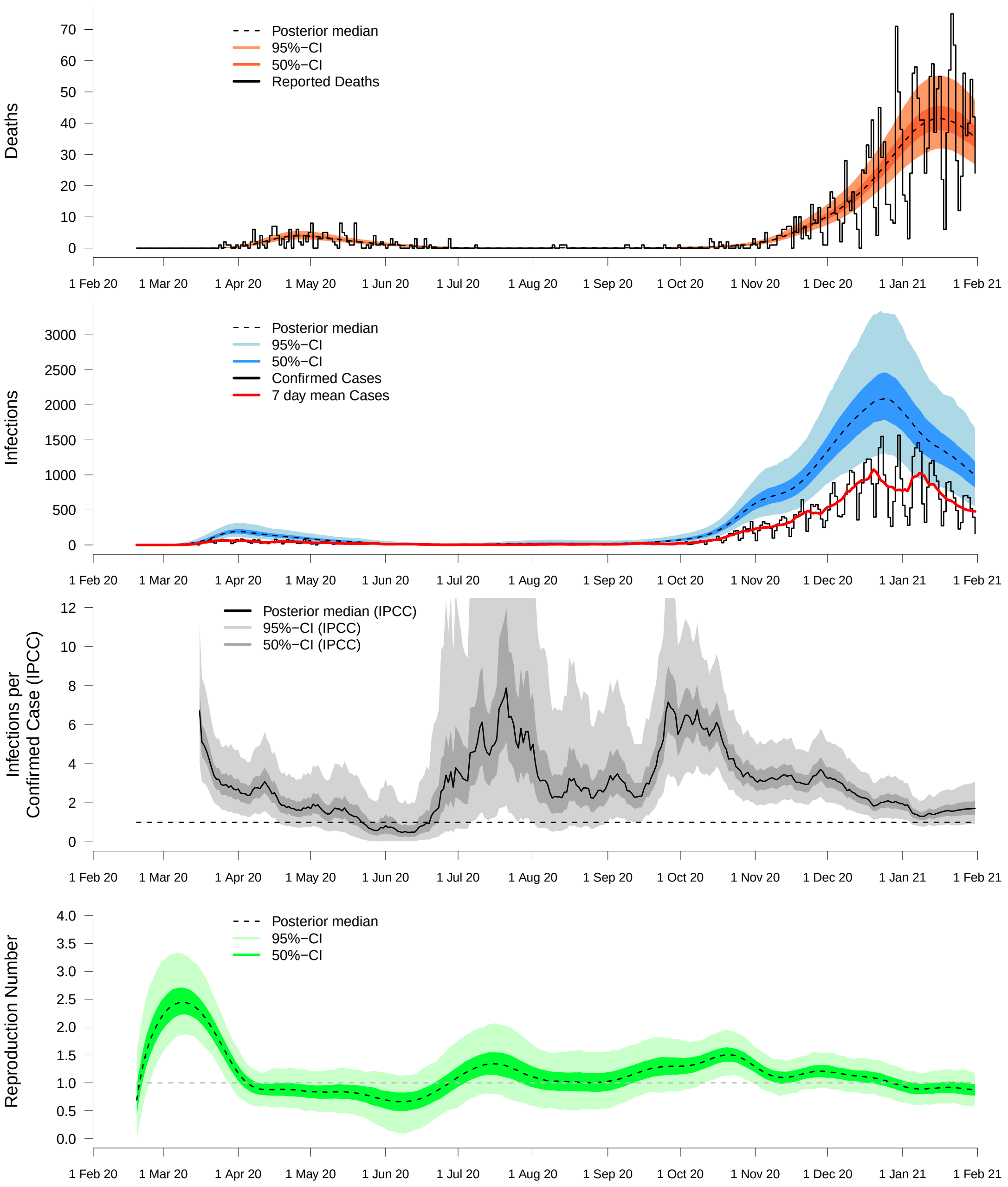

Figure S19: Results for Thuringia based on age-specific IFR estimates from Brazeau et al. [6].

S.5 Diagnostic Plots for MCMC convergence

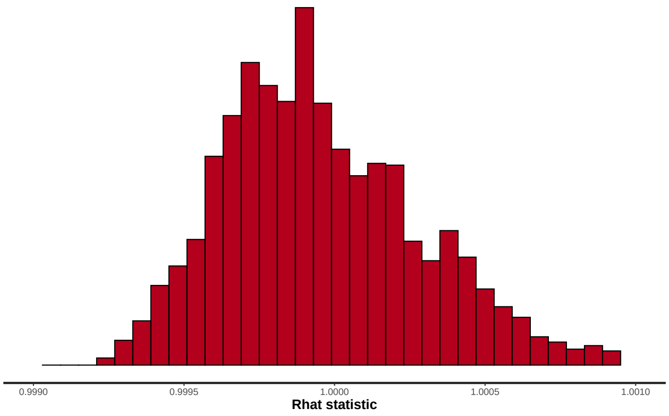

Figure S20: Histogram of Gelman-Rubin statistics (Rhat) for all parameters of the hierarchical model for Germany (values below 1.1 and close to 1 are desirable).

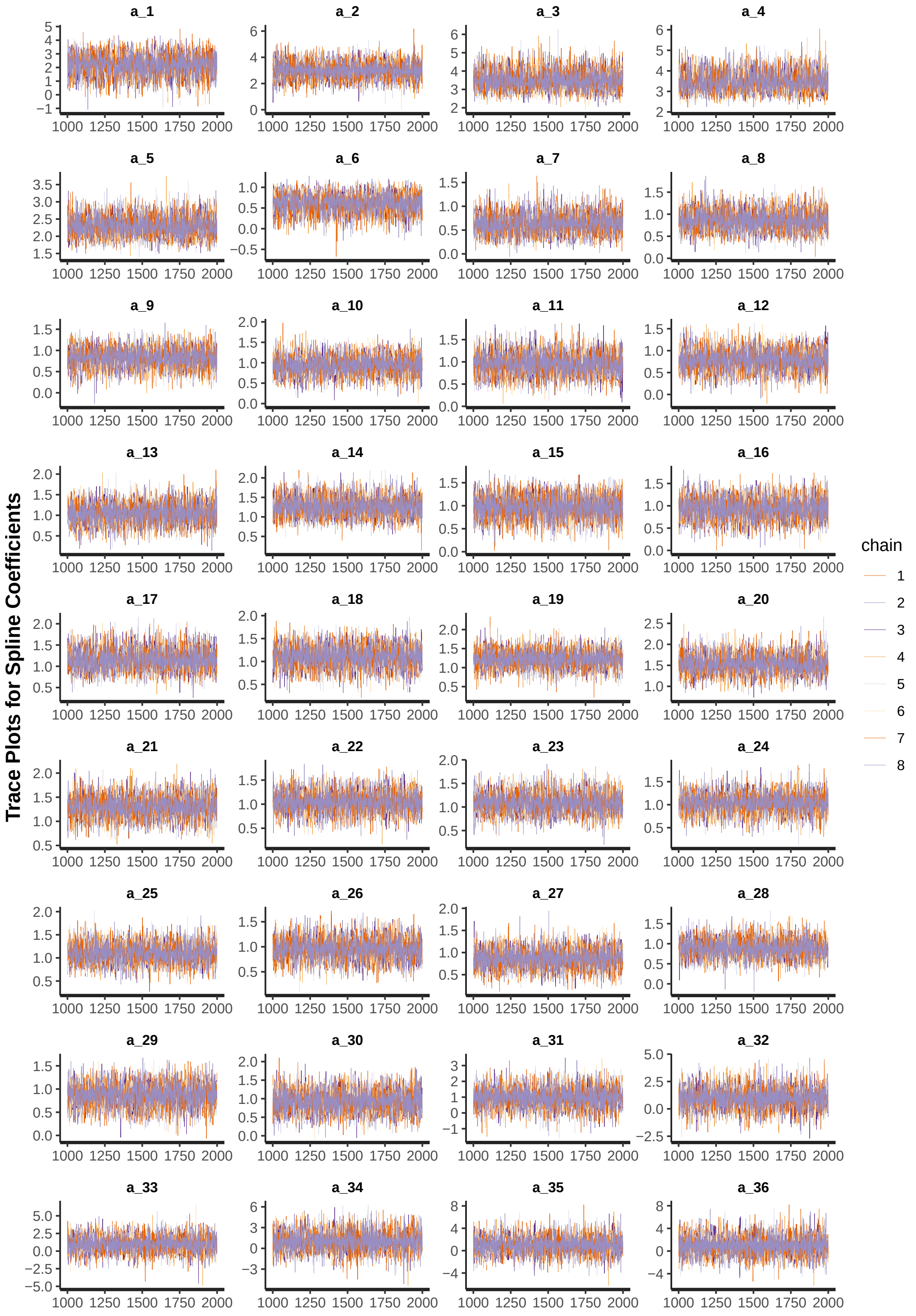

Figure S21: Trace plots of posterior samples for all spline coefficients of the hierarchical model for Germany. The eight independent chains appear to have mixed well. Trace plots for further model parameters show a similar mixing behaviour.

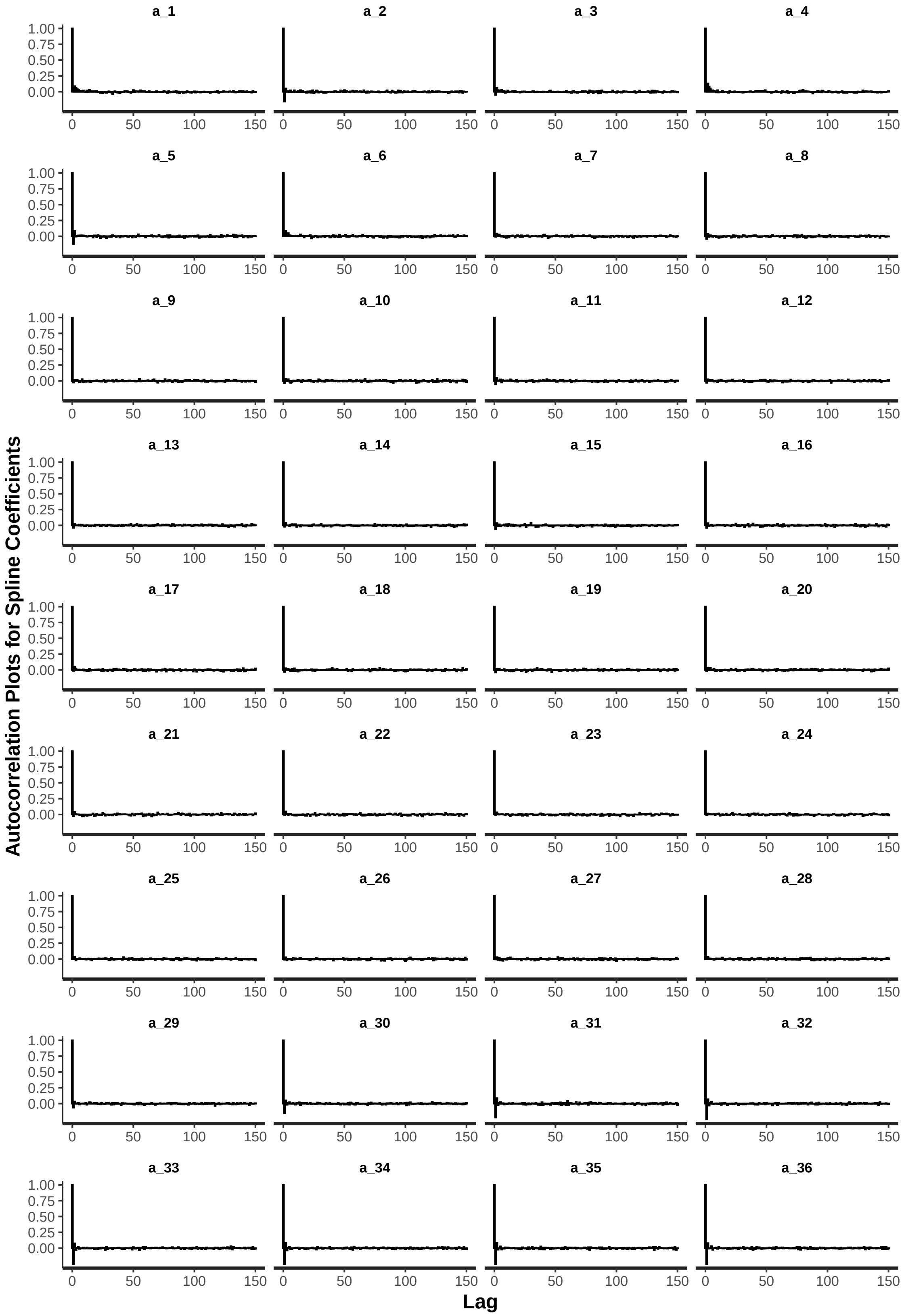

Figure S22: Autocorrelation plots for all spline coefficients of the hierarchical model for Germany, averaged over the eight independent chains. Autocorrelation plots for further model parameters are similar.

References

[1] Flaxman S, Mishra S, Gandy A, Unwin HJT, Mellan TA, Coupland H, et al. Estimating the effects of non-pharmaceutical interventions on COVID-19 in Europe. Nature. 2020;584(7820):257–261.

[2] Nishiura H, Linton NM, Akhmetzhanov AR. Serial interval of novel coronavirus (COVID-19) infections. International Journal of Infectious Diseases. 2020;93:284–286.

[3] Lauer SA, Grantz KH, Bi Q, Jones FK, Zheng Q, Meredith HR, et al. The incubation period of coronavirus disease 2019 (COVID-19) from publicly reported confirmed cases: estimation and application. Annals of Internal Medicine. 2020;172(9):577–582.

[4] O'Driscoll M, Dos Santos GR, Wang L, Cummings DA, Azman AS, Paireau J, et al. Age-specific mortality and immunity patterns of SARS-CoV-2. Nature. 2021;590(7844):140–145.

[5] Levin AT, Hanage WP, Owusu-Boaitey N, Cochran KB, Walsh SP, Meyerowitz-Katz G. Assessing the age specificity of infection fatality rates for COVID-19: systematic review, meta-analysis, and public policy implications. European Journal of Epidemiology. 2020;35:1123–1138.

[6] Brazeau N, Verity R, Jenks S, Fu H, Whittaker C, Winskill P, et al. COVID-19 infection fatality ratio: estimates from seroprevalence. Imperial College London (29-10-2020). 2020;34.
